# CT and MR image fusion of tandem and ring applicator using rigid registration in intracavitary brachytherapy planning

**DOI:** 10.1120/jacmp.v15i2.4206

**Published:** 2014-03-06

**Authors:** Arun S. Oinam, Parsee Tomar, Firuza D. Patel, Lakhwant Singh, Bhavana Rai, Amit Bahl

**Affiliations:** ^1^ Department of Radiotherapy Post Graduate Institute of Medical Education and Research Chandigarh U.T. Chandigarh India; ^2^ Physics Department Guru Nanak Dev University Institution Amritsar Punjab India

**Keywords:** autoradiograph, dwell position, MPR, intracavitary, interstitial brachytherapy, impact of registration error

## Abstract

The purpose of this study is to find the uncertainties in the reconstruction of MR compatible ring‐tandem intracavitary applicators of high‐dose rate image‐based brachytherapy treatment planning using rigid registration of 3D MR and CT image fusion. Tandem and ring reconstruction in MR image based brachytherapy planning was done using rigid registration of CT and MR applicator geometries. Verifications of registration for applicator fusion were performed in six verification steps at three different sites of tandem ring applicator set. The first site consists of three errors at the level of ring plane in (1) cranio–caudal shift (Cranial Shift) of ring plane along tandem axis, (2) antero–posterior shift (AP Shift) perpendicular to tandem axis on the plane containing the tandem, and (3) lateral shift (Lat Shift) perpendicular to the plane containing the tandem at the level of ring plane. The other two sites are the verifications at the tip of tandem and neck of the ring. The verification at the tip of tandem consists of two errors in (1) antero–posterior shift (AP Shift) perpendicular to tandem axis on the plane containing the tandem, and (2) lateral shift (Lat Shift) perpendicular to the plane containing the tandem. The third site of verification at the neck of the ring is the error due to the rotation of ring about tandem axis. The impact of translational errors from −5 mm to 5 mm in the step of 1 mm along x‐, y‐, and z‐axis and three rotational errors about these axes from −19.1° to 19.1° in the step of 3.28° on dose‐volume histogram parameters ($D_{2\text{cc}},D_{1\text{cc}},D_{0.1\text{cc}}$, and $D_{5\text{cc}}$ of bladder, rectum, and sigmoid, and $D_{90}$ and $D_{98}$ of HRCTV were also analyzed. Maximum registration errors along cranio–caudal direction was 2.2 mm (1 case), whereas the errors of 31 out of 34 cases of registration were found within 1.5 mm, and those of two cases were less than 2 mm but greater than 1.5 mm. Maximum rotational error of ring about tandem axis was 3.15° (1.1 mm). In other direction and different sites of the ring applicator set, the errors were within 1.5 mm. The impacts of registration errors on DVH parameters of bladder, rectum, and sigmoid were very sensitive to antero–posterior shift. Cranio‐caudal errors of registration also largely affected the rectum DVH parameters. Largest change of 17.95% per mm and 20.65% per mm in all the DVH parameters of all OARs and HRCTV were observed for ϕ and Ψ rotational errors as compare to other translational and rotational errors. Catheter reconstruction in MR image using rigid registration of applicator geometries of CT and MR images is a feasible technique for MR image‐based intracavitary brachytherapy planning. The applicator registration using the contours of tandem and neck of the ring of CT and MR images decreased the rotational error about tandem axis. Verification of CT MR image fusion using applicator registration which consists of six steps of verification at three different sites in ring applicator set can report all the errors due to translation and rotational shift along θ,ϕ, and Ψ. ϕ and Ψ rotational errors, which produced potential changes in DVH parameters, can be tackled using AP Shift and Lat Shift at the tip of tandem. The maximum shift was still found along the tandem axis in this technique.

PACS number: 87.55.km

## I. INTRODUCTION

The introduction of computed tomography (CT) and magnetic resonance (MR) imaging compatible applicator enables us to define the target volume and organs at risk effectively for CT and MR image‐base brachytherapy treatment planning. However, delineation of target volume in CT images is still limited due to lesser soft‐tissue contrast resolution. magnetic resonance (MR) imaging preferably $T_{2}$ weighted were recommended as a superior imaging modality for target volume and organ at risk delineation/^1‐4^ However, applicator reconstruction in magnetic resonance (MR) image is found to be difficult due to the larger thickness of slice and spacing of adjacent slices of MR image acquisition and the inabilities to visualize the proper geometry of catheters, the localization of source channel and tips of catheters in MR images. Moreover, the major challenge in MR image‐based brachytherapy is the lack of availability of dummy catheters to simulate the source positioning and poor spatial resolution for the delineation of smaller dummy source size in MR image. This inability to visualize the source channel in MR images is due to weak signal response from the applicator materials, as well as from smaller size of dummy source.^(5)^ Kirisits et al.^6^, ^7^ defined the catheters in paratransverse MR images using the back‐projection of applicator geometry reconstructed from X‐ray images and Oinam et al.^(8)^ also used the back‐projection method to reconstruct the applicator geometry in CT images. Limitation of back‐projection methods was the inability to digitize the anchor points which represent the dwell positions of library plans in the interslices points of CT and MR images. Recently multiplanar reformatted image reconstruction was introduced in different brachytherapy treatment planning systems.^9^, ^10^, ^11^, ^12^, ^13^, ^14^, ^15^, ^16^ The changes in DVH parameters calculated due to interobserver variation of applicator reconstruction using the different methods of applicator reconstructions in MR image‐based brachytherapy planning were also reported by different authors.^10^, ^11^, ^12^, ^13^ Haack et al.^(14)^ reported the interobserver reconstruction accuracy of individual catheters reconstructed using multiplanar reconstruction method and copper sulphate dummy sources in MR image‐based intracavitary brachytherapy planning. Pelloski et al.^(9)^ also used the multiplanar reconstruction method of low dose rate (LDR) brachytherapy applicators in BrachyVision TPS. If the CT data of small slice spacing is acquired, the applicator geometry can be reconstructed accurately using the information of autoradiographs. This can be utilized for the reconstruction of applicator channel in three‐dimensional MR images using the registration technique of image fusion. Presently, there are a limited number of literatures which reported about the practice of applicator reconstructions in MR image‐based brachytherapy using rigid registration of applicator geometries of CT and MR images.^(41516)^ So far, none of the studies reported about the accuracy of the definition of dwell positions using rigid registration of applicator geometries of CT and MR images in clinic. In this paper, we have attempted a method to minimize the errors in the definition of the applicator geometry and source position on 3D MR scan image in high‐dose‐rate brachytherapy treatment planning using 3D CT and MR image registration of applicator geometry. We introduced a method also to quantify and report the errors associated with 3D CT and MR image fusion of applicator geometry.

## II. MATERIALS AND METHODS

### A. Images acquisitions and contouring for brachytherapy treatment planning

Standard Nucletron CT and MR imaging compatible ring applicator (Nucletron, Veenendal, The Netherlands) of 2.6 cm and 3 cm diameter were use in this image‐based intracavitary brachytherapy implants. Vienna ring applicator (Nucletron) of 3.0 cm diameter was also used in this study. The lengths of the tandems used in this study are 4.0 cm and 6.0 cm. These implants were done with the help of MicroMaxx portable ultrasound system from Sonosite (Philips Ultrasound Inc., Seattle, WA) for proper positioning of tandem and ring applicator in the uterus and paracervical regions respectively. Abdominal obstetric gynaecological ultrasound probe C11e of 30 cm scan depth and 5‐2 MHz ultrasound frequency was used in this image‐guided brachytherapy implants. In this study, $T_{2}$ weighted turbo spin‐echo MR image sequences (TR (spin‐echo relaxation time) = 4000 ms, TE (spin‐echo elapsed time) = 112 ms) were acquired in 1.5 Tesla MR Scanner (Siemens Magnetom; Siemens AG, Munich, Germany) using a pelvic surface coil. This produces fast spin‐echo sequences with 3 mm slice thickness in different orientation of slice acquisition. As a part of EMBRACE protocol of GEC‐ESTRO recommendation guideline,^1^, ^2^ four MR image sequences consist of paratransverse, parasagittal, and paracoronal images containing the tandem and the whole ring and another transverse MR image sequence were acquired. $T_{2}$ weighted transverse MR images were use for the contouring of target volumes in Oncentra MasterPlan treatment planning system (Nucletron). Target volumes and organs at risk (OARs) were contoured on transverse MR image according to GEC‐ESTRO recommendation guideline^1^, ^2^ with the help of paratransverse, parasagittal, and paracoronal images containing the tandem and the whole ring. The OARs, contoured on the transverse MR images in this study, were sigmoid, bladder, and rectum. Gross target volume (GTV), and high risk and intermediate risk clinical target volumes (HRCTV and IRCTV) were also defined in this study.

### B. Determination of dwell position in CT and MR compatible ring applicator set

**Figure 1:**
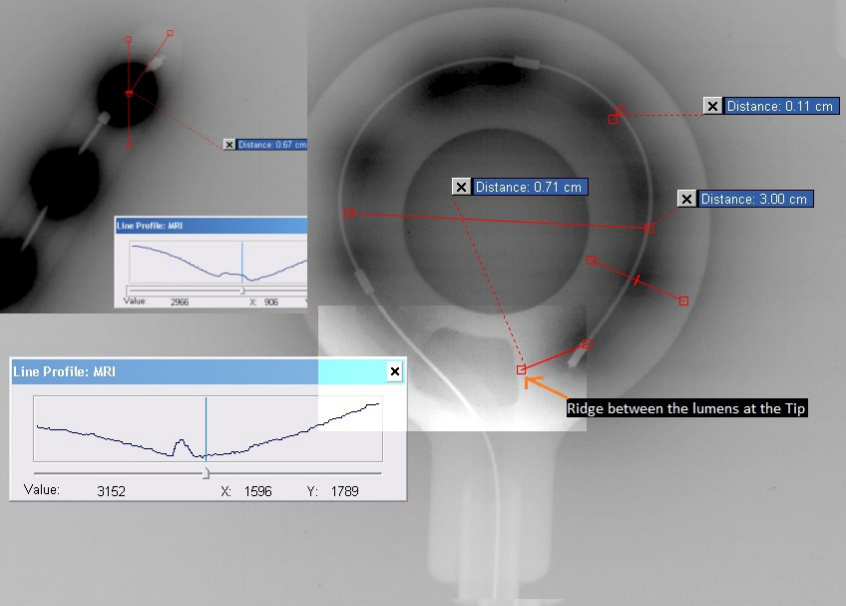
Autoradiographs of Nucletron ring applicators set for source positions identification. Figure 1(a) shows the distances of the first dwell position from the middle point of the ridge between the air channel trends (lumen of the applicator) at the tip of ring applicator as 0.71 cm. The deviation of radio‐opaque dummy source from the center of dwell position of radiation source shows as 0.11 cm. Inset figure shows the definition of first dwell position from the middle point of the ridge between the inner air channel and outer surface of applicator at the tip using the Full width at half of the maximum CT number of line profile drawn on a line passing through the center (dwell position) of the source.

Dwell position of HDR brachytherapy source inside the applicators should be coincided with the plan dwell position in the treatment planning system. The accurate definitions of dwell positions inside the applicator can be done using the autoradiography of active sources on a single film with the applicator in the same geometry. Autoradiograph of different type of applicators was taken using extended dose range KODAK EDR2 ready pack film (Eastman KODAK Company, Rochester, NY) to find out the tips and different dwell positions of radiation sources of applicators. The ring applicators and tandems used in this study were attached on this film and exposed with high‐dose‐rate (HDR) brachytherapy source (Micro Selectron HDR V3 machine; Nucletron) at different dwell positions, starting from the first dwell position. The time of exposure was optimally chosen so as to obtain a fine center of optical density for the source positioning, as well as the lumen of applicator and the surface of the applicator on the film. Then the same film with the attached applicators was exposed on kilovoltage X‐ray of 50 kV accelerating voltage and tube current of 120 mAs for 10 to 12 times to demarcate the surface and inner air channel of the applicators. After processing this film on an automatic film processor, it was scanned with a resolution of 600 dpi by an optical density scanner VIDAR VXR‐16 (VIDAR Systems Corporation, Herndon, VA) in the import workspace of ECLIPSE treatment planning system (TPS) (Varian Medical System, Palo Alto, CA) and imported as digital image communication (DICOM) format using standard mode of 1:1 scale. The center of the optical density due to the exposure by HDR radiation source was determined as the dwell position using the full width at half or tenth of the maximum value (FWHM or FWTM) of CT Hounsfield number depending on the symmetry of optical density profile. On the autoradiograph film of ring applicator, the offset value of the first dwell position of ring applicator from the middle point of the ridge between the entry and tip of air lumens of the ring applicator was measured (Fig. 1). For the intrauterine tandem applicator, the positions of the first and different dwell positions were define with respect to tip and size of the catheter (Fig. 1). Two tangents were drawn along the applicator — one at the tip and the other at the next straight portion, nearest to the curvature. Then, the distance between the tip and intersection of the two tangents were noted for the reconstruction of the same applicator in Oncentra Masterplan brachytherapy treatment planning system.

### C. Applicator reconstruction on 3D image of MRI

**Figure 2:**
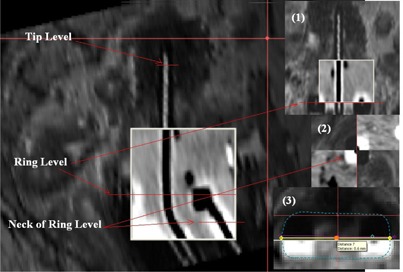
Verification of CT and MR image fusion of tandem and ring applicators on parasagittal plane with the positions of three verification sites. The inset 1, 2, 3 show the verifications of tandem and ring on paracoronal planes using dummy source, neck of ring on para‐axial plane, and the projected contours of ring applicators, reconstructed on CT and MR images, respectively.

The applicator geometries in 3D MR image were reconstructed according to CT image, fused on MR image using the rigid registration of applicator geometry. In this image fusion, the contours of tandem applicators were reconstructed separately in both CT and MR transverse images using pearls contouring tools of Oncentra MasterPlan for the preparation of CT and MR image fusion. The tips of the tandem applicators were excluded in the contouring of these tandems. In order to observe the outer surface of ring applicator in MR image, the ring applicator set and cotton used for packing in this intracavitary implants were soaked with Aquason 2000 sonography gel. Even then, the outer surface of the applicator is not seen in every slice. So the clearly observed points of applicator in MR images were use for the tandem applicator contouring in 3D MR image. Rectangular shape necks of the rings were also contoured in both CT and MR images. These reconstructed contours of tandem and ring applicators were fused interactively using rigid registration technique of Oncentra MasterPlan. The precise positioning of applicators fusion was performed by shifting and rotating these applicators of CT and MR 3D image dataset. Then the ring and tandem applicators were reconstructed on MR image using the help of corresponding coregistered CT images by digitizing the catheters on CT image through the spy glass tool and multiplanar reconstruction technique (Fig. 2). The first dwell positions on the applicators were determined in MR images (corresponding to coregistered CT images) with respect to the landmarks of the tips, curvature, and source channels of the ring applicators set using the offset values from the distal digitization point of these applicators from autoradiograph and the blend image between CT and MR images.

### D. Verification method to report the applicator registration accuracy of CT and MR images

Accuracy of applicator reconstruction in CT and MR image fusion were retrospectively analyzed for 34 cases of already done intracavitary brachytherapy application. The absolute value of the deviation of the position of applicator in MR images from the corresponding points in CT images were taken as the errors of image fusion and catheter reconstruction errors. Catheter positions of CT image were taken as the baseline geometry for image fusion. CT and MR image fusion was verified using the following six verification shifts at three different sites.

The verification of the first site is done at the level of ring plane. It consists of three errors: (1) cranio–caudal shift (Cranial Shift) of ring plane along tandem axis, (2) antero–posterior shift (AP Shift) perpendicular to tandem axis on the plane containing the tandem, and (3) lateral shift (Lat Shift) perpendicular to the plane containing the tandem.

The second site is the verification at the tip of tandem. It consists of two errors: (1) antero–posterior shift (AP Shift) perpendicular to tandem axis on the plane containing the tandem, and (2) lateral shift (Lat Shift) perpendicular to the plane containing the tandem.

The third site at the neck of the ring is the verification for the error due to the rotation of ring about tandem axis. This error can be minimized by reducing lateral shift of mean centre of the neck of ring.

The accuracy of applicator reconstruction in this CT and MR fusion technique was verified using the water dummy sources inserted in Vienna ring applicator set implants, as well as the titanium needle hole in ten cases of such image fusion. The variation of maximum deviations of reconstructed catheters from the water dummy sources on the reformatted paratransverse, parasagittal, and paracoronal MR image planes were analyzed (Fig. 2). Rotations of the ring about tandem were frequently observed and alignments using the needle holes of Vienna ring were done repeatedly after the first initial alignment of rigid registration. Later on, the rectangular shape neck of the ring, observed in both CT and MR transverse images, were contoured and accomplished while doing rigid registration. With this method, normal CT and MR compatible Nucletron ring applicator set were begun to be use in our center and the uses of water dummy sources were stopped. In case of CT and MR image fusion without water dummy sources, the projected contours of tandem and ring applicators of CT and MR images were analyzed on different reformatted para‐axial, parasagittal, and paracoronal MR images. The contours of the ring applicator which are clearly visualized on original paracoronal and parasagittal MR images were copied to the transverse MR image and edited according to the position of ring on transverse MR. Then the lateral, antero–posterior and cranio–caudal shift of ring contour of MR image from those of CT image were measured, as shown in Fig. 2. Lines passing through the mean center of the tandem were also drawn on reformatted parasagittal and paracoronal plane at the level of the tip of tandem. The maximum deviations of these lines from those of CT image were measured using spy glass tool of Oncentra Masterplan for each of the CT and MR image fusion plan. These deviations were analyzed for 24 plans of such image fusions. Similarly the deviation of mean center of the rectangular shape catheter at the neck of the ring on reformatted para‐axial MR image from those of CT image were measured (Fig. 2).

### E. Impact of image registration errors on DVH

Bladder, rectum, sigmoid, and HRCTV contours of a typical patient of GEC ESTRO EMBRACE protocol were considered to find out the impact of registration errors on dose‐volume histogram parameter in this study. To quantify the changes in dose‐volume histogram parameters due to rgistration errors in applicator reconstruction of brachytherapy planning, known errors in catheter reconstructions have to be introduced in applicator coordinate system. The coordinate points of reconstructed catheters of Oncentra Treatment Planning system were define in MR image coordinate system which is used as primary image in image registration. In order to introduce the known errors in applicator coordinate system, MR image coordinate system was transformed into applicator coordinate system using an autorotation program developed in MATLAB software version 7.7 (The MathWorks, Natick, MA) and determines the three rotational angles and three translational shifts. The equations used in this autorotation program were

(1) $$\left(\begin{array}{l} {\text{xSt}}_{i}\\ {\text{ySt}}_{i}\\ {\text{zSt}}_{i} \end{array}\right)=R_{\text{xyz}}(\theta ,\phi ,\psi )\cdot \left(\begin{array}{l} {(x}_{i}{‐o}_{x})\\ {(y}_{i}{‐o}_{y})\\ {(z}_{i}{‐o}_{z}) \end{array}\right)$$

where $R_{\text{XYZ}}(\theta ,\phi ,\psi )$ is the rotation matrix as a function of θ,ϕ,Ψ, which are rotating about y‐, z‐, and x‐axes, respectively. θ is the rotation of applicator set about tandem axis (y). ϕ is the rotation about an axis (z) through the center of the ring on the tandem plane and perpendicular to the tandem axis. Ψ is the rotation about an axis (x) through the center of the ring on the ring plane and perpendicular to the plane containing the tandem applicator. ${\text{xSt}}_{i},{\text{ySt}}_{i}$, and ${\text{zSt}}_{i}$ are the ith coordinates of applicator in applicator coordinate system, and $x_{i},y_{i}$, and $z_{i}$ are the coordinates of applicators with origin of $o_{x},o_{y}$, and $o_{z}$ of applicator in MR image coordinate system. After the determination of θ,ϕ, and Ψ using three orthogonal coordinate points of applicator set within the tolerance limit of 0.3 mm in two steps of autorotation programs consisting of coarse rotation of 1 mm tolerance and fine rotation of 0.3 mm tolerance limits, different systematic errors were introduced in six degrees of freedom (x‐, y‐, z‐axes and θ,ϕ, and Ψ rotational angles) in rigid registration. Then the inverse rotation were performed using the already determined angles of rotations (θ,ϕ, and Ψ) to transform into MR image coordinate system. The equation used in the reverse rotation for the introduction of translational error is

(2) $$\left(\begin{array}{l} x_{i}\\ y_{i}\\ z_{i} \end{array}\right)=R_{\text{xyz}}^{\text{inv}}(\theta ,\phi ,\psi )\cdot \left(\begin{array}{l} {\text{xSt}}_{i}+\Delta x\\ {\text{ySt}}_{i}+\Delta y\\ {\text{zSt}}_{i}+\Delta z \end{array}\right)$$

For rotational error introduction along the rotation about y‐axis, the following equations are use in our program:

(3) $$\left(\begin{array}{l} {\text{xSt}}_{i}^{'}\\ {\text{ySt}}_{i}^{'}\\ {\text{zSt}}_{i}^{'} \end{array}\right)=R_{\text{xyz}}(\Delta \theta ,0,0)\cdot \left(\begin{array}{l} {\text{xSt}}_{i}\\ {\text{ySt}}_{i}\\ {\text{zSt}}_{i} \end{array}\right)$$

(4) $$\left(\begin{array}{l} x_{i}\\ y_{i}\\ z_{i} \end{array}\right)=R_{\text{xyz}}^{\text{inv}}(\theta ,\phi ,\psi )\cdot \left(\begin{array}{l} {\text{xSt}}_{i}^{'}\\ {\text{ySt}}_{i}^{'}\\ {\text{zSt}}_{i}^{'} \end{array}\right)+\left(\begin{array}{l} o_{x}\\ o_{y}\\ o_{z} \end{array}\right)$$

The errors introduced in applicator coordinate system were ranges from −5 mm to 5 mm in the step of 1 mm along tandem, vertical, and horizontal axis of tandem and rotational errors (Δθ) which ranges from −19.10° to 19.10° in the step of 3.28°. In cases of rotational errors (Δϕ and ΔΨ) introduction along ϕ and Ψ rotational angles, $R_{\text{XYZ}}(0,\Delta \phi ,0)$ and $R_{\text{XYZ}}(0,0,\Delta \psi )$ rotational matrices were use. The changes in dose to 2 cc, 1 cc, 0.1 cc, and 5 cc volumes of bladder, rectum, and sigmoid ($D_{2\text{cc}},D_{1\text{cc}},D_{0.1\text{cc}}$, and $D_{5\text{cc}}$ of bladder, rectum, and sigmoid), 90% and 98% volume of HRCTV ($D_{90}$ and $D_{98}$) and volume of HRCTV receiving 90% percent of prescribed dose $\left(V_{90}\right)$ due to these errors were analyzed for 66 applicator reconstructions to validate the action limit for images registration.

## III. RESULTS & DISCUSSION

Figure 1 shows the autoradiographs of intracavitary ring applicator set for source positions identification. This depicts both the dwell positions of HDR radiation source, as well as the applicator geometries of ring and tandem applicators. The distances of the first dwell position from the center of the ridge between the air lumens at the tip of different ring applicators were 7.1 mm and 6.7 mm, respectively, for normal Nucletron rings of 3 cm and 2.6 cm diameter, and offset of 9.5 mm was found for Vienna Nucletron ring of 3.0 cm diameter. The center of the ridge was specially chosen for the digitization of the ring catheter to minimize the uncertainty of digitization of the tips of applicators. Similarly the distances of the first dwell positions of tandem applicators from the same centers of the ridge between the inner air lumen and the outer surface of different tandems at the tip were found as 6.9 mm for both 4 cm and 6 cm long tandems. The distance of the cranial surface of ring applicator from the source channel were found as 6.2 mm, 6.1 mm, and 6.2 mm for normal Nucletron ring of 3.0 cm and 2.6 cm diameters and Vienna Nucletron ring of 3.0 cm diameter (Table 1). The HDR miniature source was positioned at the center of the inner air trend of normal Nucletron ring applicator of 3.0 cm diameter, whereas the position of radio‐opaque dummy was shifted toward the outer wall of the inner trend by 0.11 cm.

Dummy markers to simulate the position of the source in 3D CT image were use by Pelloski et al.^(9)^ in the multiplanar reconstruction of brachytherapy applicators in BrachyVision TPS (Varian Medical System). But in our experience, the determination of the tips and the source positions with dummy marker were still a challenging task and we found uncertainties due to the inability of accurate determinations of tips by the limited slice spacing in CT data acquisition and source position using dummy marker. Reconstruction of catheter was done easily using MPR method with the help of the rotation of the axes of 3D CT image, zoom in, and distance measuring tools of Oncentra MasterPlan TPS and the corresponding autoradiograph informations instead of dummy markers. In this applicator reconstruction, paracoronal and parasagittal original MR images were not used even though these images generate good clear boundary of applicator. This is due to the inability of the acquisition of the MR image along the tandem axis and the movement of patient during longer data acquisition time between two different sequences for accurate localization of source channel.

**Table 1:** The dwell positions of different applicators in autoradiograph

		*Outer Dimension (mm)*		*Distance Between Source Channels and Outer Applicator Surface (mm)*		
*Type of Applicator*	*Model Ring / Tandem*	*Width (Lateral) / Diameter*	*Length (Cranio‐caudal)*	*To cranial surface*	*To lateral surface*	*Distance of First Dwell Position from the Tip* ^a^ *(mm)*
Ring	3.0 cm diameter (Normal ring)	43.0 mm (Normal ring)	—	6.2 mm	6.0 mm	7.1 mm
	2.6 cm diameter (Normal ring)	40.0 mm (Normal ring)	—	6.1 mm	6.4mm	6.7 mm
	3.0 cm diameter (Vienna ring)	43.0 mm (Vienna ring)	—	6.2 mm	6.3mm	9.5 mm
Tandem	4 cm	—	40.0 mm	—	—	6.9 mm
	6 cm	—	60.0 mm	—	—	6.9 mm

^a^ Distance of first dwell position from the middle point of the ridge between the inner air lumens at the tip of ring catheter or the inner air lumen and outer applicator surface at the tip of tandem

**Figure 3:**
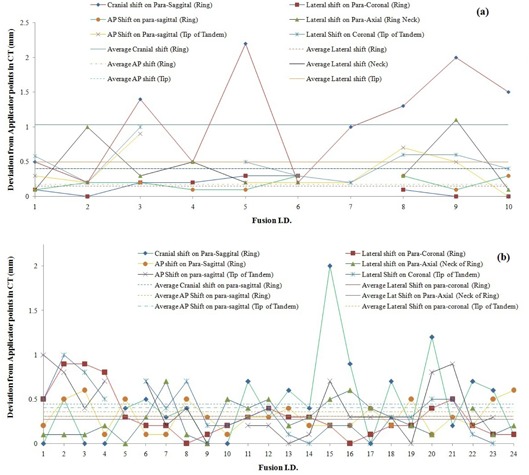
Variation of six verification parameters for CT and MR image fusions at three different sites: ring level, tip level of tandem, and neck level of ring: (a) using water dummy source of MR image (10 cases of CT and MR image fusions), and (b) without water dummy source of MR image (24 cases of CT and MR image fusions).

In our experience, maximum errors were found along the cranio–caudal direction and the rotation of ring about tandem axis while using rigid registration of CT MR image fusion for applicator reconstruction. Haack et al.^(14)^ also reported four variations of the interobserver reconstruction accuracy of catheters in MR image‐based intracavitary brachytherapy planning using BrachyVision TPS. The variations were reported in terms of antero–posterior, lateral, and longitudinal translational shifts and rotation of the ring about tandem axis. In our experience the errors must be reported for a complete set of applicator implants and at different sites of applicator rather than for individual applicator and single site. This can be done by verifying at three different sites of this ring applicator set instead of reporting individual applicators in their study (Fig. 2). The variation of six different shifts of reconstructed catheter in MR image using coregistered CT image from the water‐filled dummy sources inside the applicators of MR images for ten cases of reconstructions are depicted in Fig. 3(a). Table 2 also shows the statistics of errors (Cranial Shift, AP shift, and Lat Shift at the ring level) of reconstructed catheter from the position of water dummy sources at the level of ring on reformatted paracoronal and parasagittal planes. The maximum deviation of 2.2 mm (1 case) on paracoronal/sagittal plane was found along the cranial direction, as compared to those of lateral shift on paracoronal plane and AP shift on parasagittal plane. In case of rotation of ring applicator about tandem axis, maximum Lat Shift of neck of ring from water dummy sources was 1.1 mm (3.15°). Maximum values of AP Shift on parasagittal plane and Lat Shift on paracoronal plane of MR image from CT image were also below 0.5 mm. The maximum error was found along the cranial direction, with an average of 1.03 mm (SD=0.72 mm) and 0.45 mm (SD=0.46 mm) for the verification of catheter reconstructions with and without water‐filled dummy sources, respectively (Fig. 3 and Table 2). Haack et al.^(14)^ also showed the same maximum variations in the direction of longitudinal axis of tandem and in the direction of ring rotation about tandem axis. Two cases of fusion were beyond the action limit of 1.5 mm, whereas the remaining registrations were within 1.5 mm (Fig. 3(a)). This occurred due to the unnoticeable shift of tandem applicator of MR image from CT image along the tandem axis while doing the applicator registration. Table 2 also shows the verification of reconstructed catheters of 24 cases of MR image‐based brachytherapy plans without water dummy sources. The maximum Cranial Shift of ring contour of MR from those of CT along the tandem axison paracoronal or parasagittal plane was 2 mm at the ring level. Only one case of catheter reconstruction had a Cranial Shift greater than 1.5 mm (Fig. 3(b)). This method was done to quantify the error along cranio–caudal, lateral, and antero–posterior shift of ring from that of CT image in this study. Such a procedure cannot be performed in routine practice due to time‐consuming procedures of copying the contours of ring applicator and editing on transverse MR images, but the gross error can be verified interactively in ECS coordinate system of Oncentra Master Plan TPS without this contour. Maximum values of AP Shift on parasagittal and Lat Shift on paracoronal plane at the level of ring were 0.6 mm and 0.9 mm, respectively. At the tip of tandem, maximum values of AP Shift on parasagittal plane and Lat Shift on paracoronal plane were 1.0 mm and 1.0 mm, respectively, whereas those of Lat Shift at the neck of the ring resulted due to the rotation of ring about tandem axiswas found as 0.7 mm. This rotation was corrected later on in our applicator registration by aligning the contours of the rectangular shape neck of the ring (Fig. 2) and reduced the average shift to 0. 27 mm from 0.46 mm with water dummy source (Fig. 3 and Table 2).

**Figure 4:**
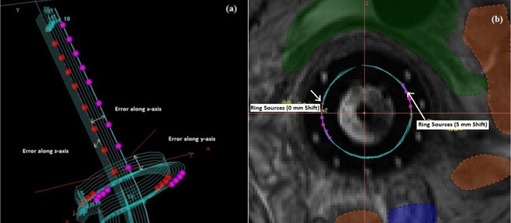
Catheters (a) reconstructed with the introduction of translational errors (1 mm to 5 mm in the step of 1 mm) along tandem axis(x), horizontal axis(y), and vertical axis(z). Red color spheres represent the original dwell position of reconstructed applicators and purple spheres represent the same dwell positions with 5 mm error introduced to original reconstructed catheters in horizontal axis(x). Catheters (b) reconstructed with the introduction of rotational errors (0° to 19.1° in the step of 3.28°) along θ. Red color spheres represent the original dwell position of reconstructed applicators and purple spheres represent the same dwell positions with 19.1° error introduced to original reconstructed catheters in horizontal axis(x).

**Figure 5:**
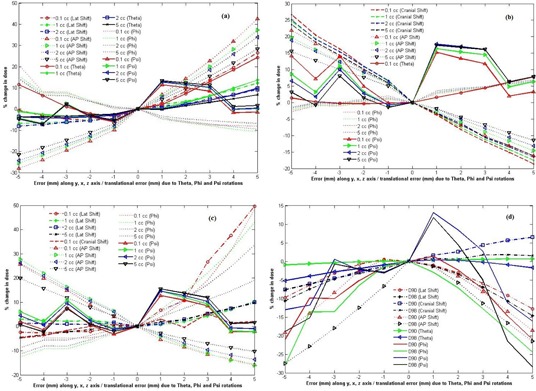
Impact of registration errors oarameters of different OARs and HRCTV: % change of DVH parameters of bladder (a), rectum (b), sigmoid (c), and HRCTV (d) due to translational and rotational errors, respectively, from those of original reconstruction.

In our center, we analyzed the impact of registration errors on DVH parameters for a patient using the autorotation program incorporating the above mathematical (1), (4). In this analysis, multiple reconstructions were performed for a single application by introducing the different systematic errors in applicator coordinate system (Fig. 4) to find out the effects of systematic errors on DVH parameters. Figure 5 shows the variations of DVH parameters from those of original reconstructed catheters of ring and tandem applicators with different systematic errors ranges from −5 mm to 5 mm in the step of 1 mm along three axes of horizontal, vertical, and parallel to tandem as translational errors and rotational errors ranges from −19.1° to 19.1° in the step of 3.82° about tandem y‐axis, z‐axis, and x‐axis.

**Table 2:** Reconstruction accuracy of applicators using CT/MRI fused images with and without water filled dummy sources

		*At Ring Level*			*At the Neck Level of Ring*	*At the Tip Level of Tandem*	
*Method (No. of Cases)*		*Cranial Shift* ^a^ *(mm) (Paracoronal)*	*AP Shift* ^b^ *(mm) (Parasagittal)*	*Lat Shift* ^c^ *(mm) (Paracoronal)*	*Lat Shift* ^c^ *(mm (°)) (Para‐axial)*	*AP Shift* ^b^ *(mm) (Parasagittal)*	*Lat Shift* ^c^ *(mm) (Paracoronal)*
Water filled dummy (10)	Average	1.03	0.18	0.15	0.46 (1.32)	0.40	0.50
					(1.32)		
	St. Dev.	0.72	0.09	0.12	0.38	0.29	0.25
					(1.09)		
	Maximum	2.20	0.30	0.30	1.10	0.90	1.00
					(3.15)		
Without water filled dummy (24)	Average	0.45	0.31	0.31	0.27	0.40	0.36
					(0.77)		
	St. Dev.	0.46	0.17	0.25	0.20	0.31	0.27
					(0.57)		
	Maximum	2.00	0.60	0.90	0.70	1.00	1.00
					(2.00)		
Total (34)	Average	0.63	0.26	0.28	0.32	0.39	0.40
					(0.91)		
	St. Dev.	0.61	0.24	0.16	0.26	0.30	0.27
					(0.74)		
	Maximum	2.20	0.90	0.60	1.10	1.00	1.00
					(3.15)		

^a^ Cranial Shift: Perpendicular shift to the plane of ring on sagittal/coronal plane along the tandem axis.

^b^ AP Shift: Perpendicular shift to the axisof tandem on sagittal plane containing tandem.

^c^ Lat Shift: Lateral shift perpendicular to the plane containing tandem axison paracoronal / para‐axial plane.

**Figure 6:**
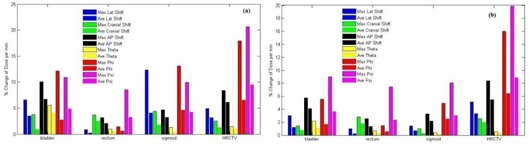
Impact of registration errors in terms of % absolute changes of DVH parameters per mm for different OARs and HRCTV: (a) *%* absolute change of dose per mm to 0.1 cc volumes of all OARs and HRCTV $D_{90}$ due to translational and rotational errors; (b) % absolute change of dose per mm to 2 cc volume of all OARs and HRCTV $D_{98}$ due to translational and rotational errors.

In Fig. 5(a), the maximum percent changes in dose to 0.1 cc $\left(D_{0.1\text{cc}}\right)$, 1 cc $\left(D_{1\text{cc}}\right)$ and 2 cc $\left(D_{2\text{cc}}\right)$ volume of bladder due to the introduction of errors from −5 mm to 5 mm along x‐axis from the same dose‐volume histogram (DVH) parameters of original reconstructed applicators were, respectively, as 26.6%, 12.23%, and 9.19%. $D_{0.1\text{cc}}$ was very sensitive to error introduced. Percent changes of dose to 5 cc $\left(D_{5\text{cc}}\right)$ volume of bladder varied from −7.78% to 5.58%. All the DVH parameters of bladder varied linearly with respect to systematic errors introduced along x‐, y‐, and z‐axis. Maximum variation of 42.54% for $D_{0.1\text{cc}}$ was found due to the introduction of 5 mm systematic error along z‐axis, as compare to those errors introduced along x‐ and y‐axes in the decreasing order. The variations all the DVH parameters due to the errors along y‐axis ranged from −7.6% to 2.66%. When the rotational errors were introduced along θ,ϕ, and Ψ, all the DVH parameters varied in nonlinear pattern except for $D_{1\text{cc}},D_{2\text{cc}}$, and $D_{5\text{cc}}$ due to rotational errors along 9 (Fig. 5(a)). Rotational errors along ϕ and Ψ result a large percent change of all DVH parameters of all OARs and HRCTV, as compare to those changes due to other systematic errors (Fig. 6). Applicator registrations of this study were done using the tandem contours of CT and MR images, the maximum values of both AP Shift and Lat Shift at the tip of tandem and the ring level were within 1 mm. The systematic errors due to rotational error along ϕ and Ψ due to 6 cm long tandem were within 1° and hence the maximum impacts on DVH parameters for Ψ and ϕ rotational errors were within 5% and 2%, respectively. The maximum (average) absolute percent changes of dose per mm of $D_{0.1\text{cc}}$ of bladder due to systematic errors along the lateral, cranio–caudal, and antero–posterior directions were 6.62% (3.54%), 3.79% (0.86%), and 10.11% (6.73%), respectively, and for those changes due to the rotation (θ) about tandem was 5.59% (4.08%) per mm (Fig. 6(a)). In case of $D_{2\text{cc}}$, the maximum (average) absolute changes of dose per mm due to the errors along lateral, cranio–caudal, and antero–posterior directions were 2.99% (1.22%), 1.41% (0.73%), and 5.75% (4.12%), respectively, whereas the error of rotation (9) about tandem results the maximum (average) value of 2.21% (1.04%) (Fig. 6(b)).

Figure 5(b) shows the linear variation of rectum DVH parameters ($D_{0.1\text{cc}},D_{1\text{cc}},D_{2\text{cc}}$, and $D_{5\text{cc}}$) due to systematic translational (along cranio–caudal and antero–posterior directions) and rotational errors (along θ,ϕ, and Ψ) with respect to those of original reconstruction. Maximum ranges of variations from −18.59% to 26.6% and −16.11% to 21.89% were found for $D_{0.1\text{cc}}$ of rectum due to cranio–caudal and antero–posterior errors, respectively, whereas those due to lateral error were from −0.61% to 2.90%. When the rotational errors were introduced along θ,ϕ, and Ψ, both the errors along θ and ϕ produced the approximate linear changes of all DVH parameters (within the variation ranges from 0% to 4.04% and −1.45 to 6.8%, respectively, for $D_{2\text{cc}}$), whereas all the DVH parameters due to the error along Ψ (rotation about x‐axis) were found as nonlinear changes (Fig. 5(b)). Maximum (average) absolute change of dose per mm for $D_{0.1\text{cc}}$ and $D_{2\text{cc}}$ were found maximum as 3.69% (2.53%) and 2.82% (1.75%), respectively, for the systematic error along cranio–caudal shift, as compare to those of other systematic errors, except ϕ and Ψ rotational errors (Fig. 6(a)).

In case of sigmoid (Fig. 5(c)), all the DVH parameters varied linearly with systematic errors along lateral (‐4.56% to 10.47%), cranial‐caudal (‐4.70% to 5.28%), AP shift (‐16.10% to 27.65%), and θ rotation (‐2.05% to 2.05%). $D_{1\text{cc}}$ and $D_{5\text{cc}}$ due to ϕ rotational error also varied linearly within the ranges from −12.5% to 42.95% and −8.74% to 19.01%, respectively, whereas $D_{0.1\text{cc}}$ of sigmoid due to lateral error and all the DVH parameters due the rotational error along Ψ varied in nonlinear pattern. $D_{0.1\text{cc}}$ due to lateral errors varies up to 49.58% from those of original reconstruction geometry of applicator. Maximum (average) absolute percent change of dose per mm due to lateral error was 12.36% (4.09%) per mm for $D_{0.1\text{cc}}$, whereas those of other DVH parameters were found within 3.28% (2.19%) per mm, except those of error along ϕ and Ψ (Fig. 6). All the DVH parameters of sigmoid were very sensitive to AP Shift, ϕ, and Ψ rotational errors.

Most of the DVH parameters of HRCTVs varied in nonlinear pattern, as shown in Fig. 5(d). Maximum variation range from ‐ 27.49% to 0.49% was found for AP shift error as compare to other ranges from −10.6% to 0.51% and −7.65% to 6.49% of Lat Shift and cranio–caudal shift errors, respectively. In case of rotational error, ϕ and Ψ rotational errors produced the variation in DVH parameters from −27.93% to 1.35% and −28.39% to 11.77%, respectively. Percent absolute change of dose per mm for HRCTV $D_{98}$ and $D_{90}$ were within the maximum values of 8.39% per mm and 8.37% per mm and average values of 6.14% per mm and 5.51% per mm, respectively. HRCTV $V_{90}$ was not affected by the systematic errors introduced in this study. Maximum changes of HRCTV $V_{90}$ were found within 2.09%. The changes in DVH parameters calculated due to interobserver variation of applicator reconstruction using rigid registration methods were also reported by Tanderup et al.^(15)^ They reported the changes of DVH parameters of bladder and rectum by 5%‐6% per mm displacement of applicator in ant–post direction. For the other directions and other DVH parameters, the changes were lesser than 4% per mm. Our data were well congruent as that of the mean change of dose per mm of bladder and rectum which were within 4% per mm due to lateral and cranio–caudal shifts. We found a very large change of 10.92% per mm and 12.16% per mm for ϕ and Ψ rotational errors in all DVH parameter of all OARs ($D_{0.1\text{cc}},D_{1\text{cc}},D_{2\text{cc}}$, and $D_{5\text{cc}}$) and HRCTV ($D_{90}$ and $D_{98}$) as compare to other translational and rotational errors. It is reasonably correct that Tanderup et al. report only the error along θ for rotational error. As the alignment was done using tandem contours of CT and MR images, the rotational errors along ϕ and Ψ do not happened frequently. However, a small change of applicator registration error of 1 mm at the tip of tandem will result in 1° error in ϕ and Ψ rotation about an origin at the center of the ring and hence a large change in all DVH parameters can happen (5, 6). These can be taken care by checking and reporting the accuracy of applicator registration at the tip along AP Shift and Lat Shift within 1.0 mm action limit. Cranial Shift largely affects the DVH parameters of rectum only (Fig. 6) as compare to bladder, sigmoid, and HRCTV. Action limits of 1.0 mm to 2.0 mm will produce the increase in variation of dose of rectum from those of original reconstruction ranges from 6.7% to 10.9%. The limitation of this study is that the impacts of registration error on the changes of DVH parameters were considered only for a single patient. These impacts will be better quantified if a large population of patient data were utilized.

## IV. CONCLUSIONS

To our knowledge, our study is one of the new procedures for reporting the registration errors of CT MR fusion using rigid registration method for applicator reconstruction and to analyze the impact of registration errors oarameters in image‐based brachytherapy planning. Image‐based brachytherapy planning requires an accurate definition of dwell position of radiation source with respect to the tips of the applicators othe MR images. Choosing the ridge between the lumens at the tip of the ring applicator as reference point for dwell position definition decreased the uncertainty of digitization of catheter. The applicator geometries of micro‐Selectron HDR brachytherapy can be successfully reconstructed in treatment planning system using the rigid registration of applicators of CT and MR images and the information of applicator geometries from autoradiographs. The applicator registration of CT and MR images using the contours of tandem and neck of the ring decreased the rotational error about tandem axis. The reconstruction accuracy of applicators was achieved within the action limit of 1.5 mm in this CT MR Image fusion technique. We recommended a verification method of CT MR image fusion using applicator registration which consists of six steps of verification at three different sites in ring applicator set for a perfect fusion. Rotational errors along ϕ and Ψ rotation angles, which produced large changes in DVH parameters, can be tackled using AP Shift and Lat Shift at the tip of tandem. The maximum shift was found along the tandem axis in this technique.

## Supporting information

Supplementary Material

Supplementary Material

Supplementary Material

Supplementary Material

Supplementary Material

Supplementary Material

Supplementary Material
