# Supplementary figures and images for: CT and MR image fusion of tandem and ring applicator using rigid registration in intracavitary brachytherapy planning
Source: J Appl Clin Med Phys. 2014 Mar 6;15(2):191–204. doi: 10.1120/jacmp.v15i2.4206 (PMC5875474; doi:10.1120/jacmp.v15i2.4206)

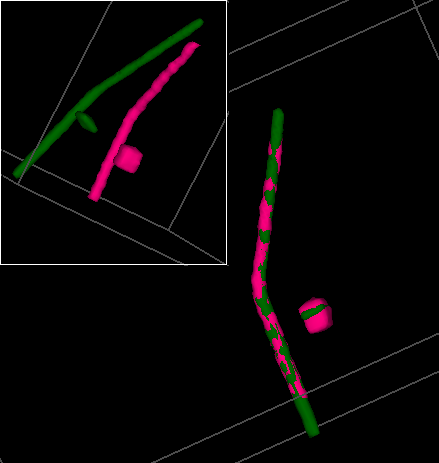

Supplement: Supplementary file 1 — Supplementary Material [file ACM2-15-191-s001.TIF]

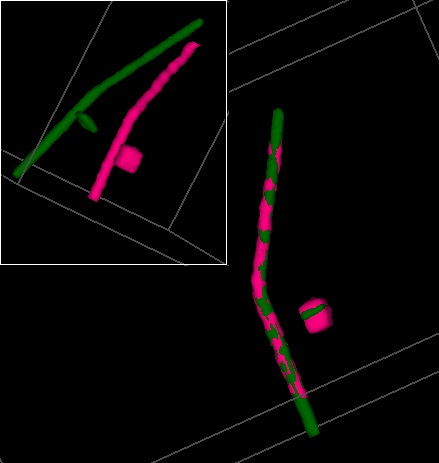

Supplement: Supplementary file 2 — Supplementary Material [file ACM2-15-191-s002.jpg]

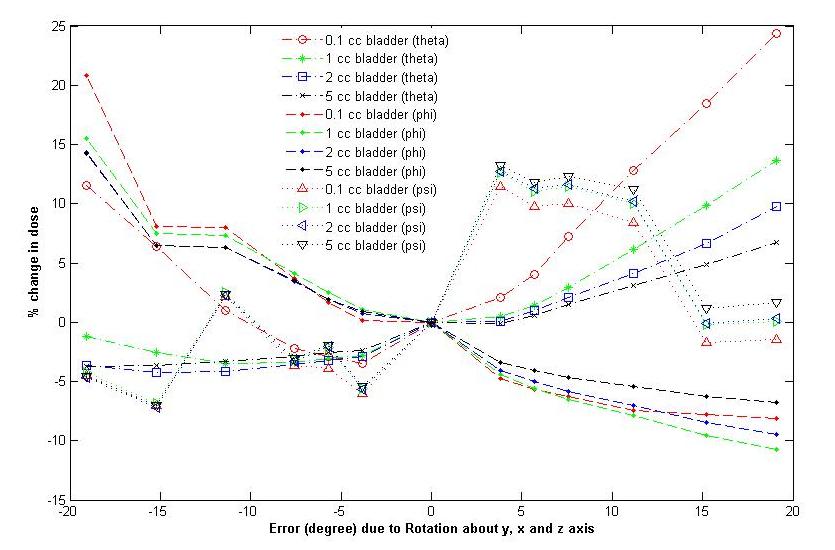

Supplement: Supplementary file 3 — Supplementary Material [file ACM2-15-191-s003.jpg]

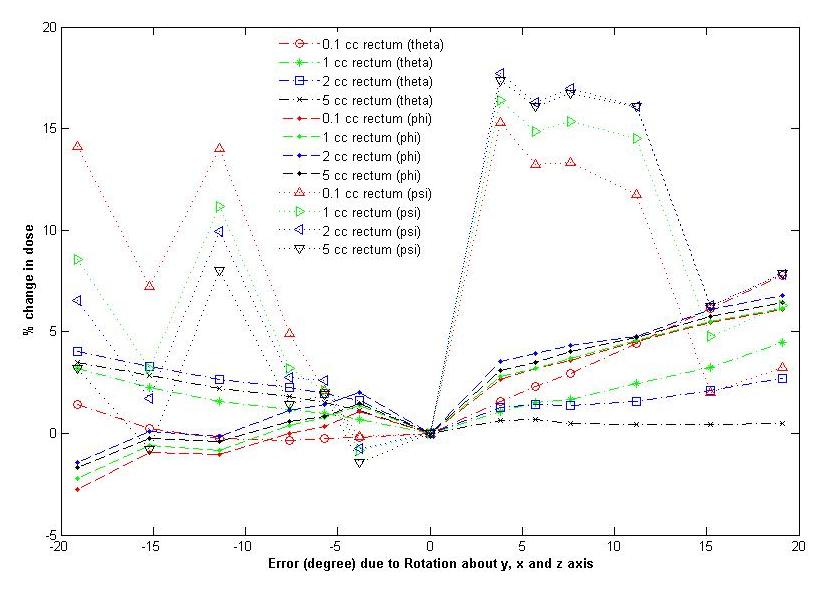

Supplement: Supplementary file 4 — Supplementary Material [file ACM2-15-191-s004.jpg]

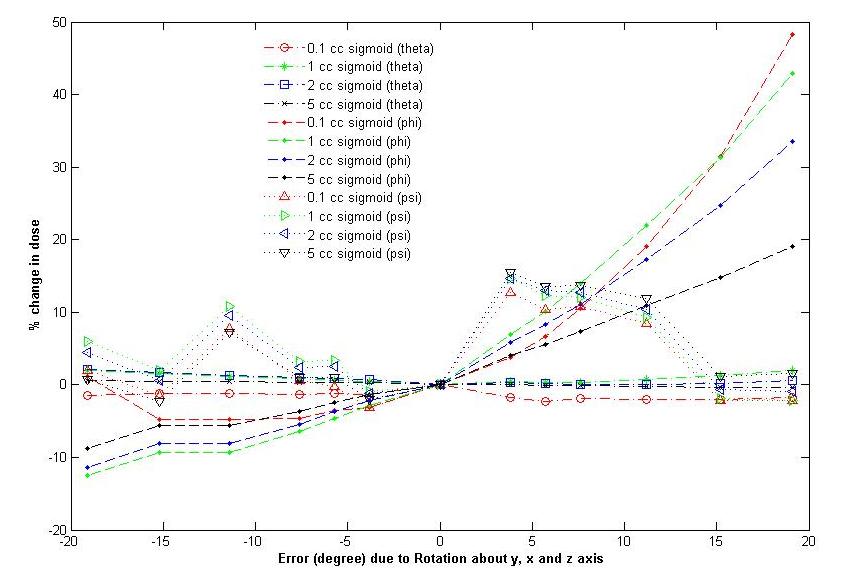

Supplement: Supplementary file 5 — Supplementary Material [file ACM2-15-191-s005.jpg]

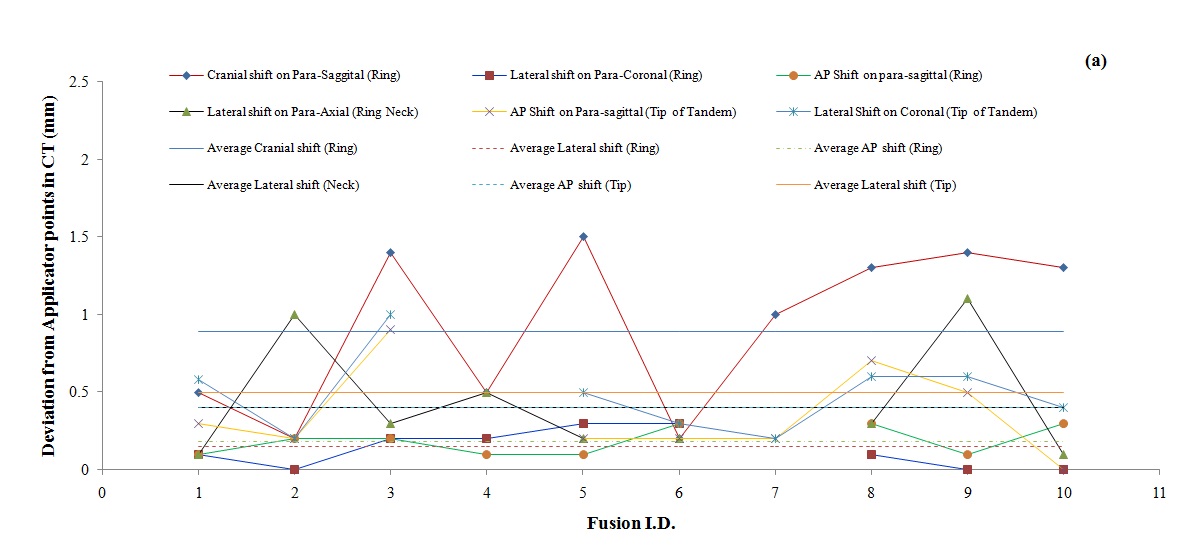

Supplement: Supplementary file 7 — Supplementary Material [file ACM2-15-191-s007.jpg]
