# Supplementary material for: CT and MR image fusion of tandem and ring applicator using rigid registration in intracavitary brachytherapy planning
Source: J Appl Clin Med Phys. 2014 Mar 6;15(2):191–204. doi: 10.1120/jacmp.v15i2.4206 (PMC5875474; doi:10.1120/jacmp.v15i2.4206)
Supplement: Supplementary file 6 — Supplementary Material [file ACM2-15-191-s006.doc]

CT and MR image fusion of tandem and ring applicator using rigid registration in Intracavitary Brachytherapy Planning

**ABSTRACT**

**PURPOSE:** The purpose of this study is to find the uncertainties in the reconstruction of MR compatible ring-tandem intracavitary applicators of high dose rate Image based Brachytherapy treatment planning using rigid registration of 3D MR and CT image fusion.

**MATERIALS AND METHODS:** Tandem and ring reconstruction in MR Image based Brachytherapy planning was done using rigid registration of CT and MR applicator geometries. Verifications of registration for applicator fusion were performed in 6 verification steps at 3 different sites of tandem ring applicator set. The first site consists of 3 errors at the level of ring plane in (1) cranio-caudal shift (Cranial Shift) of ring plane along tandem axis, (2) antero-posterior shift (AP Shift) perpendicular to tandem axis on the plane containing the tandem, (3) lateral shift (Lat Shift) perpendicular to the plane containing the tandem at the level of ring plane. The other two sites are the verifications at the tip of tandem and neck of the ring. The verification at the tip of tandem consists of 2 errors in (1) antero-posterior shift (AP Shift) perpendicular to tandem axis on the plane containing the tandem, (2) lateral shift (Lat Shift) perpendicular to the plane containing the tandem. The third site of verification at the neck of the ring is the error due to the rotation of ring about tandem axis. The impact of translational errors from -5 mm to 5 mm in the step of 1 mm along x, y and z-axis and 3 rotational errors about these axes from -19.1° to 19.1° in the step of 3.28° on Dose Volume Histogram parameters (D_2cc_, D_1cc_, D_0.1cc_ and D_5cc_ of Bladder, Rectum and Sigmoid and D_90_ and D_98_ of HRCTV) were also analysed.

**RESULTS:** Maximum registration errors along cranio-caudal direction was 2.2 mm (1 case) whereas the errors of 31 out of 34 cases of registration were found within 1.5 mm and those of 2 cases were lesser than 2 mm but greater than 1.5 mm. Maximum rotational error of ring about tandem axis was 2.94° (1.1 mm). In other direction and different sites of the ring applicator set, the errors were within 1.5 mm. The impacts of registration errors on DVH parameters of bladder, rectum and sigmoid were very sensitive to Antero-posterior shift. Cranio-caudal errors of registration also largely affected the rectum DVH parameters. Largest change of 17.95% per mm and 20.65% per mm in all the DVH parameters of all OARs and HRCTV were observed for φ and ψ rotational errors as compare to other translational and rotational errors.

**CONCLUSION:** Catheter reconstruction in MR image using rigid registration of applicator geometries of CT and MR images is a feasible technique for MR Image based Intracavitary Brachytherapy planning. The applicator registration using the contours of tandem and neck of the ring of CT and MR images decreased the rotational error about tandem axis. Verification of CT MR image fusion using applicator registration which consists of 6 steps of verification at 3 different sites in ring applicator set can report all the errors due to translation and rotational shift along θ, φ and ψ. φ and ψ rotational errors which produced a potential changes in DVH parameters can be tackled using AP Shift and Lat Shift at the tip of tandem. The maximum shift was still found along the tandem axis in this technique.

**Key Words:** Auto-radiograph, dwell position, MPR, intracavitary, interstitial brachytherapy, impact of registration error.

1. **INTRODUCTION**

The introduction of Computed Tomography (CT) and Magnetic Resonance (MR) Imaging compatible applicator enables us to define the target volume and organs at risk effectively for CT and MR Image base Brachytherapy treatment planning. However, delineation of target volume in CT images is still limited due to lesser soft tissue contrast resolution. Magnetic Resonance (MR) imaging preferably T_2_ weighted were recommended as a superior imaging modality for target volume and organ at risk delineation.^(1-4)^ However, applicator reconstruction in Magnetic Resonance (MR) image is found to be difficult due to the larger thickness of slice and spacing of adjacent slices of MR image acquisition and the inabilities to visualize the proper geometry of catheters, the localization of source channel and tips of catheters in MR images. Moreover, the major challenge in MR Image based Brachytherapy is the lack of availability of dummy catheters to simulate the source positioning and poor spatial resolution for the delineation of smaller dummy source size in MR Image. This inability to visualize the source channel in MR images is arised due to weak signal response from the applicator materials as well as from smaller size of dummy source.^(5)^  Kirisits et al^(6-8)^ defined the catheters in para-transverse MR images using the Backprojection of applicator geometry reconstructed from x-ray images. Limitation of Backprojection methods was the inability to digitize the anchor points which represent the dwell positions of library plans in the inter-slices points of CT and MR images. Recently multi-planar reformatted image reconstruction was introduced in different brachytherapy treatment planning systems.^(9-16)^ The changes in DVH parameters calculated due to inter-observer variation of applicator reconstruction using the different methods of applicator reconstructions in MR Image base Brachytherapy planning were also reported by different authors.^(10-13)^ Haack et al.^(14)^ reported the inter-observer reconstruction accuracy of individual catheters reconstructed using multi-planar reconstruction method and cupper sulphate dummy sources in MR Image based Intracavitary Brachytherapy planning. Pelloski et al.^(9)^ also used the multi-planar reconstruction method of LDR (Low Dose Rate) brachytherapy applicators in Brachy Vision TPS. If the CT data of small slice spacing is acquired, the applicator geometry can be reconstructed accurately using the information of auto-radiographs. This can be utilized for the reconstruction of applicator channel in 3 Dimensional MR images using the registration technique of image fusion. Presently, there is limited number of literatures which reported about the practice of applicator reconstructions in MR Image base Brachytherapy using rigid registration of applicator geometries of CT and MR images.^(4, 15, 16)^ So far none of the studies reported about the accuracy of the definition of dwell positions using rigid registration of applicator geometries of CT and MR images in clinic. In this paper we have attempted a method to minimize the errors in the definition of the applicator geometry and source position on 3D MR scan image in high dose rate brachytherapy treatment planning using 3D CT and MR Image registration of applicator geometry. We introduced a method also to quantify and report the errors associated with 3D CT and MR image fusion of applicator geometry.

1. **METHOD AND MATERIALS**
2. **Images acquisitions and Contouring for brachytherapy treatment planning**

Standard Nucletron CT and MR Imaging compatible ring applicator (Nucletron, Veenendal, The Netherland) of 2.6 cm and 3 cm diameter were used in this Image based Intracavitary Brachytherapy implants. Vienna ring applicator (Nucletron, Veenendal, The Netherland) of 3.0 cm diameter was also used in this study. The lengths of the tandems used in this study are 4.0 cm and 6.0 cm. These implants were done with the help of MicroMaxx® portable ultrasound system from Sonosite^TM^ Ultrasound Inc., WA, USA for proper positioning of tandem and ring applicator in the uterus and paracervical regions respectively. Abdominal Obstetric Gynaecological ultrasound probe C11e of 30 cm scan depth and 5-2 MHz ultrasound frequency was used in this image guided Brachytherapy Implants. In this study, T_2_ weighted turbo spin-echo MR image sequences [TR (spin-echo relaxation time) = 4000 ms, TE (spin-echo elapsed time) = 112 ms] were acquired in 1.5 Tesla MR Scanner (Siemens Magnetom; Siemens AG, Munich, Germany) using a pelvic surface coil. This produces fast spin-echo sequences with 3 mm slice thickness in different orientation of slice acquisition. As a part of EMBRACE protocol of GEC-ESTRO recommendation guideline, ^(1, 2)^ four MR image sequences consist of para-transverse, para-sagittal and para-coronal images containing the tandem and the whole ring and another transverse MR image sequence were acquired. T_2_ weighted transverse MR images were used for the contouring of target volumes in Oncentra MasterPlan treatment planning system (Nucletron, Netherland). Target volumes and organs at risk (OARs) were contoured on transverse MR image according to GEC-ESTRO recommendation guideline ^(1, 2)^ with the help of para-transverse, para-sagittal and para-coronal images containing the tandem and the whole ring. The OARs, contoured on the transverse MR images in this study were sigmoid, bladder and rectum. Gross Target volume (GTV), High Risk and Intermediate Risk Clinical Target volumes (HRCTV and IRCTV) were also defined in this study.

1. **Determination of dwell position in CT and MR compatible ring applicator set**

Dwell position of HDR brachytherapy source inside the applicators should be coincided with the plan dwell position in the Treatment Planning System. The accurate definitions of dwell positions inside the applicator can be done using the autoradiography of active sources on a single film with the applicator in the same geometry. Auto-radiograph of different type of applicators was taken using extended dose range KODAK EDR2 ready pack film (Eastman KODAK Company, Rochester, NY, USA) to find out the tips and different dwell positions of radiation sources of applicators. The ring applicators and tandems, used in this study were attached on this film and exposed with High Dose Rate (HDR) Brachytherapy source (Micro Selectron HDR V3 machine, Nucletron, Netherland) at different dwell positions starting from the first dwell position. The time of exposure was optimally chosen so as to obtain a fine centre of optical density for the source positioning as well as the lumen of applicator and the surface of the applicator on the film. Then the same film with the attached applicators was exposed on kilovoltage x-ray of 50 kV accelerating voltage and tube current of 120 mAs for 10 to 12 times to demarcate the surface and inner air channel of the applicators. After processing this film on an automatic film processor, it was scanned with a resolution of 600 dpi by an optical density scanner VIDAR VXR-16 (VIDAR Systems Corporation, Herndon, VA, USA) in the import workspace of ECLIPSE treatment planning system (TPS) of Varian Medical System, Palo Alto, CA, USA and imported as digital image communication (DICOM) format using standard mode of 1:1 scale. The centre of the optical density due to the exposure by HDR radiation source was determined as the dwell position using the full width at half or tenth of the maximum value (FWHM or FWTM) of CT Hounsfield number depending on the symmetry of optical density profile. On the auto-radiograph film of ring applicator, the offset value of the first dwell position of ring applicator from the middle point of the ridge between the entry and tip of air lumens of the ring applicator was measured (figure 1). For the intrauterine tandem applicator, the positions of the first and different dwell positions were defined with respect to tip and size of the catheter (figure 1). Two tangents were drawn along the applicator – one at the tip and the other at the next straight portion, nearest to the curvature. Then, the distance between the tip and intersection of the two tangents were noted down for the reconstruction of the same applicator in Oncentra Masterplan Brachytherapy treatment planning system (Nucletron, Netherland).

1. **Applicator reconstruction on 3D image of MRI**

The applicator geometries in 3D MR image were reconstructed according to CT image, fused on MR image using the rigid registration of applicator geometry. In this image fusion, the contours of tandem applicators were reconstructed separately in both CT and MR transverse images using pearls contouring tools of Oncentra MasterPlan for the preparation of CT and MR image fusion. The tips of the tandem applicators were excluded in the contouring of these tandems. In order to observe the outer surface of ring applicator in MR image, the ring applicator set and cotton used for packing in this intracavitary implants were soaked with ultrasound lubricating gelly Aquason 2000 sonography gel. Even then, the outer surface of the applicator is not seen in every slices. So the clearly observed points of applicator in MR images were used for the tandem applicator contouring in 3D MR image. Rectangular shape necks of the rings were also contoured in both CT and MR images. These reconstructed contours of tandem and ring applicators were fused interactively using rigid registration technique of Oncentra MasterPlan. The precise positioning of applicators fusion was performed by shifting and rotating these applicators of CT and MR 3D Image dataset. Then the ring and tandem applicators were reconstructed on MR image using the help of corresponding co-registered CT images by digitizing the catheters on CT image through the spy glass tool and multiplanar reconstruction technique (Figure 2). The first dwell positions on the applicators were determined in MR images (corresponding to co-registered CT images) with respect to the landmarks of the tips, curvature and source channels of the ring applicators set using the offset values from the distal digitization point of these applicators from auto-radiograph and the blend image between CT and MR images.

1. **Verification Method to Report the Applicator Registration Accuracy of CT and MR images**

Accuracy of applicator reconstruction in CT and MR image fusion were retrospectively analysed for 34 cases of already done intracavitary Brachytherapy application. The absolute value of the deviation of the position of applicator in MR images from the corresponding points in CT images were taken as the errors of Image fusion and catheter reconstruction errors. Catheter positions of CT image were taken as the baseline geometry for image fusion. CT and MR image fusion was verified using the following 6 verification shifts at 3 different sites.

- The verification of the first site is done at the level of ring plane. It consists of 3 errors as (1) cranio-caudal shift (Cranial Shift) of ring plane along tandem axis, (2) antero-posterior shift (AP Shift) perpendicular to tandem axis on the plane containing the tandem, (3) lateral shift (Lat Shift) perpendicular to the plane containing the tandem.
- The second site is the verification at the tip of tandem. It consists of 2 errors as (1) antero-posterior shift (AP Shift) perpendicular to tandem axis on the plane containing the tandem and (2) lateral shift (Lat Shift) perpendicular to the plane containing the tandem.
- The third site at the neck of the ring is the verification for the error due to the rotation of ring about tandem axis. This error can be minimized by reducing lateral shift of mean centre of the neck of ring.

The accuracy of applicator reconstruction in this CT and MR fusion technique was verified using the water dummy sources inserted in Vienna ring applicator® set implants as well as the titanium needle hole in 10 cases of such image fusion. The variation of maximum deviations of reconstructed catheters from the water dummy sources on the reformatted para-transverse, para-sagittal and para-coronal MR image planes were analysed (figure 2). Rotations of the ring about tandem were frequently observed and alignments using the needle holes of Vienna Ring were done repeatedly after the first initial alignment of rigid registration. Later on, the rectangular shape neck of the ring, observed in both CT and MR transverse images were contoured and accomplished while doing rigid registration. With this method, normal CT and MR compatible Nucletron ring applicator set were begun to use in our centre and the uses of water dummy sources were stopped further. In case of CT and MR image fusion without water dummy sources, the projected contours of tandem and ring applicators of CT and MR images were analysed on different reformatted para-axial, para-sagittal and para-coronal MR images. The contours of the ring applicator which are clearly visualized on original para-coronal and para-sagittal MR images were copied to the transverse MR image and edited according to the position of ring on transverse MR. Then the lateral, antero-posterior and cranio-caudal shift of ring contour of MR image from those of CT image were measured as shown in figure 2. Lines passing through the mean centre of the tandem were also drawn on reformatted para-sagittal and para-coronal plane at the level of the tip of tandem. The maximum deviations of these lines from those of CT image were measured using spy glass tool of Oncentra Masterplan for each of the CT and MR image fusion plan. These deviations were analysed for 24 plans of such image fusions. Similarly the deviation of mean centre of the rectangular shape catheter at the neck of the ring on reformatted para-axial MR image from those of CT image were measured (figure 2).

1. **Impact of Image Registration Errors on DVH**

Bladder, rectum, sigmoid and HRCTV contours of a typical patient of GEC ESTRO EMBRACE protocol was considered to find out the impact of registration errors on Dose Volume Histogram parameter in this study. To quantify the changes in Dose Volume Histogram parameters due to registration errors in applicator reconstruction of brachytherapy planning, known errors in catheter reconstructions have to be introduced in applicator co-ordinate system. The co-ordinate points of reconstructed catheters of Oncentra Treatment Planning system were defined in MR image co-ordinate system which is used as primary image in Image Registration. In order to introduce the known errors in applicator co-ordinate system, MR image co-ordinate system was transformed into applicator co-ordinate system using an autorotation program developed in MATLAB software version 7.7 of MathWorks^TM^, US and determines the three rotational angles and three translational shifts. The equations used in this auto-rotation program were

$\begin{matrix} \left(\begin{matrix} xSt_{i} \\ ySt_{i} \\ zSt_{i} \end{matrix}\right) & = & R_{XYZ}\left(\theta,\varphi,\psi\right) \end{matrix}.\left(\begin{matrix} (x_{i}-o_{x}) \\ (y_{i}-o_{y}) \\ (z_{i}-o_{z}) \end{matrix}\right)$ ----------------------------------- (1)

Where $R_{XYZ}\left(\theta,\varphi,\psi\right)$ is the rotation matrix as a function of $\theta$, $\varphi$ and $\psi$which are rotate about y, z and x-axes respectively. $\theta$is the rotation of applicator set about tandem axis (y). $\varphi$is the rotation about an axis (z) through the centre of the ring on the tandem plane and perpendicular to the tandem axis. Ψ is the rotation about an axis (x) through the centre of the ring on the ring plane and perpendicular to the plane containing the tandem applicator. xSt_i_, ySt_i_ and zSt_i_ are the i^th^ co-ordinates of applicator in applicator co-ordinate system and x_i_, y_i_ and z_i_ are the co-ordinates of applicators with origin of o_x_, o_y_ and o_z_ of applicator in MR image co-ordinate system. After the determination of $\theta$, $\varphi$ and $\psi$using three orthogonal coordinate points of applicator set within the tolerance limit of 0.3 mm in two steps of auto-rotation programs consists of coarse rotation of 1 mm tolerance and fine rotation of 0.3 mm tolerance limits, different systematic errors were introduced in 6 degrees of freedom (X, Y, Z-axes and$\theta$, $\varphi$ and $\psi$rotational angles) in Rigid registration. Then the inverse rotation were performed using the already determined angles of rotations ($\theta$, $\varphi$ and $\psi$) to transform into MR image co-ordinate system. The equation used in the reverse rotation for the introduction of translational error is

$\begin{matrix} \left(\begin{matrix} x_{i} \\ y_{i} \\ z_{i} \end{matrix}\right) & = & R_{XYZ}^{inv}\left(\theta,\varphi,\psi\right) \end{matrix}.\left(\begin{matrix} xSt_{i}+\Delta x \\ ySt_{i}+\Delta y \\ zSt_{i}+\Delta z \end{matrix}\right)$+ $\left(\begin{matrix} o_{x} \\ o_{y} \\ o_{z} \end{matrix}\right)$ -------------------------------(2)

For rotational error introduction along the rotation about Y-axis, the following equations are used in our program

$\begin{matrix} \left(\begin{matrix} xSt_{i}^{'} \\ ySt_{i}^{'} \\ zSt_{i}^{'} \end{matrix}\right) & = & R_{XYZ}\left(\Delta\theta,0,0\right) \end{matrix}.\left(\begin{matrix} xSt_{i} \\ ySt_{i} \\ zSt_{i} \end{matrix}\right)$ ------------------------------------------------(3)

$\begin{matrix} \left(\begin{matrix} x_{i} \\ y_{i} \\ z_{i} \end{matrix}\right) & = & R_{XYZ}^{inv}\left(\theta,\varphi,\psi\right) \end{matrix}.\left(\begin{matrix} xSt_{i}^{'} \\ ySt_{i}^{'} \\ zSt_{i}^{'} \end{matrix}\right)$+ $\left(\begin{matrix} o_{x} \\ o_{y} \\ o_{z} \end{matrix}\right)$ --------------------------------------(4)

The errors introduced in applicator co-ordinate system were ranges from -5 mm to 5 mm in the step of 1 mm along tandem, vertical and horizontal axis of tandem and rotational errors ($\Delta\theta$) which ranges from -19.10 to 19.10 degrees in the step of 3.28 degrees. In cases of rotational errors ($\Delta\varphi$and $\Delta\Psi$) introduction along $\varphi$ and $\Psi$rotational angles, $R_{XYZ}\left(0,\Delta\varphi,0\right)$ and $R_{XYZ}\left(0,0,\Delta\psi\right)$ rotational matrices were used. The changes in dose to 2 cc, 1 cc, 0.1 cc and 5 cc volumes of bladder, rectum and sigmoid (D_2cc_, D_1cc_, D_0.1cc_ and D_5cc_ of Bladder, Rectum and Sigmoid), 90% and 98% volume of HRCTV (D_90_ and D_98_) and volume of HRCTV receiving 90% percent of prescribed dose (V_90_) due to these errors were analyzed for 66 applicator reconstructions to validate the action limit for images registration.

1. **RESULTS AND DISCUSSIONS**

Figure 1 shows the auto-radiographs of intracavitary ring applicator set for source positions identification. This depicts both the dwell positions of HDR radiation source as well as the applicator geometries of ring and tandem applicators. The distances of the first dwell position from the centre of the ridge between the air lumens at the tip of different ring applicators were 7.1 mm and 6.7 mm respectively for normal Nucletron rings of 3 cm and 2.6 cm diameter and offset of 9.5 mm was found for Vienna Nucletron ring of 3.0 cm diameter. The centre of the ridge was specially chosen for the digitization of the ring catheter to minimize the uncertainty of digitization of the tips of applicators. Similarly the distances of the first dwell positions of tandem applicators from the same centres of the ridge between the inner air lumen and the outer surface of different tandems at the tip were found as 6.9 mm for both 4 cm and 6 cm long tandems. The distance of the cranial surface of ring applicator from the source channel were found as 6.2 mm, 6.1 mm and 6.2 mm for Normal Nucletron ring of 3.0 cm and 2.6 cm diameters and Vienna Nucletron ring of 3.0 cm diameter (Table 1). The HDR miniature source was positioned at the centre of the inner air trend of Normal Nucletron ring applicator of 3.0 cm diameter whereas the position of radio-opaque dummy was shifted toward the outer wall of the inner trend by 0.11 cm.

Dummy marker to simulate the position of the source in 3D CT image were used by Pelloski et al.^(9)^ in the multi-planar reconstruction of brachytherapy applicators in Brachy Vision TPS (Varian Medical System, Charlottesville, VA, USA). But in our experience, the determination of the tips and the source positions with dummy marker were still a challenging task and found uncertainties due to the inability of accurate determinations of tips by the limited slice spacing in CT data acquisition and source position using dummy marker. Reconstruction of catheter was done easily using MPR method with the help of the rotation of the axes of 3D CT image, zoom in and distance measuring tools of Oncentra MasterPlan TPS and the corresponding auto-radiograph informations instead of dummy markers. In this applicator reconstruction, para-coronal and para-sagittal original MR images were not used eventhough these images generate good clear boundary of applicator. This is due to the inability of the acquisition of the MR image along the tandem axis and the movement of patient during longer data acquisition time in between two different sequences for accurate localization of source channel.

In our experience, maximum errors were found along the cranio-caudal direction and the rotation of ring about tandem axis while using rigid registration of CT MR image fusion for applicator reconstruction. Haack et al.^(14)^ also reported 4 variations of the inter-observer reconstruction accuracy of catheters in MR Image base Intracavitary Brachytherapy planning using Brachy Vision TPS of Varian Medical System, Charlottesville, VA, USA. The variations were reported in terms of Antero-posterior, Lateral and Longitudinal translational shifts and rotation of the ring about tandem axis. In our experience the errors must be reported for a complete set of applicator implants and at different sites of applicator rather than for individual applicator and single site. This can be done by verifying at 3 different sites of this ring applicator set instead of reporting individual applicators in their study (figure 2). The variation of 6 different shifts of reconstructed catheter in MR image using co-registered CT image from the water filled dummy sources inside the applicators of MR images for 10 cases of reconstructions are depicted in figure 3a. Table 2 also shows the statistics of errors (Cranial Shift, AP shift and Lat Shift at the ring level etc.) of reconstructed catheter from the position of water dummy sources at the level of ring on reformatted para-coronal and para-sagittal planes. The maximum deviation of 2.2 mm (1 case) on para-coronal/sagittal plane was found along the cranial direction as compared to those of lateral shift on para-coronal plane and AP shift on para-sagittal plane. In case of rotation of ring applicator about tandem axis, maximum Lat Shift of neck of ring from water dummy sources was 1.1 mm (3.15 degrees). Maximum values of AP Shift on para-sagittal plane and Lat Shift on para-coronal plane of MR image from CT image were also below 0.5 mm. The maximum error was found along the cranial direction with an average of 1.03 mm (SD = 0.72 mm) and 0.45 mm (SD = 0.46 mm) for the verification of catheter reconstructions with and without water filled dummy sources respectively (figure 3 and table 2). Haack et al.^(14)^ also showed the same maximum variations in the direction of longitudinal axis of tandem and in the direction of ring rotation about tandem axis. Two cases of fusion were beyond the action limit of 1.5 mm whereas the remaining registrations were within 1.5 mm (figure 3a). This is occurred due to the unnoticeable shift of tandem applicator of MR image from CT image along the tandem axis while doing the applicator registration. Table 2 also shows the verification of reconstructed catheters of 24 cases of MR Image base Brachytherapy plans without water dummy sources. The maximum Cranial Shift of ring contour of MR from those of CT along the tandem axis on para-coronal or para-sagittal plane was 2 mm at the ring level. Only one case of catheter reconstruction had a cranial shift greater than 1.5 mm (figure 3b). This method was done to quantify the error along Cranio-caudal, lateral and antero-posterior shift of ring from that of CT image in this study. Such a procedure cannot be performed in routine practice due to time consuming procedures of copying the contours of ring applicator and editing on transverse MR images, but the gross error can be verified interactively in ECS co-ordinate system of OncentraMaster Plan TPS without this contour. Maximum values of AP Shift on para-sagittal and Lat Shift on para-coronal plane at the level of ring were 0.6 mm and 0.9 mm respectively. At the tip of tandem, maximum values of AP Shift on para-sagittal plane and Lat Shift on para-coronal plane were 1.0 mm and 1.0 mm respectively whereas those of Lat Shift at the neck of the ring resulted due to the rotation of ring about tandem axis was found as 0.7 mm. This rotation was taken care later on in our applicator registration by aligning the contours of the rectangular shape neck of the ring (figure 2) and reduced the average shift to 0.27 mm from 0.46 mm with water dummy source (figure 3 and table 2).

In our centre, we analyzed the impact of registration errors on DVH parameters for a patient using the auto-rotation program incorporating the above mathematical equations 1-4. In this analysis, multiple reconstructions were performed for a single application by introducing the different systematic errors in applicator co-ordinate system (figure 4) to find out the effects of systematic errors on DVH parameters. Figure 5 shows the variations of DVH parameters from those of original reconstructed catheters of ring and tandem applicators with different systematic errors ranges from -5 mm to 5 mm in the step of 1 mm along three axes of horizontal, vertical and parallel to tandem as translational errors and rotational errors ranges from -19.1° to 19.1° in the step of 3.82° about tandem (y) axis, z-axis and x-axis.

In figure 5a, the maximum percent changes in dose to 0.1 cc (D_0.1cc_), 1 cc (D_1 cc_) and 2 cc (D_2 cc_) volume of bladder due to the introduction of errors from -5 mm to 5 mm along x-axis from the same Dose Volume Histogram (DVH) parameters of original reconstructed applicators were respectively as 26.6%, 12.23% and 9.19%. D_0.1cc_ was very sensitive to error introduced. Percent changes of dose to 5 cc (D_5 cc_) volume of bladder varied from -7.78 % to 5.58 %. All the DVH parameters of bladder varied linearly with respect to systematic errors introduced along x, y and z-axis. Maximum variation of 42.54% for D_0.1cc_ was found due to the introduction of 5 mm systematic error along z-axis as compare to those errors introduced along x and y-axes in the decreasing order. The variations all the DVH parameters due to the errors along y-axis ranged from -7.6 % to 2.66 %. When the rotational errors were introduced along θ, φ and ψ, all the DVH parameters varied in non-linear pattern except for D_1 cc_, D_2 cc_ and D_5 cc_ due to rotational errors along θ (Figure 5a). Rotational errors along φ and ψ result a large percent change of all DVH parameters of all OARs and HRCTV as compare to those changes due to other systematic error (figure 6). Applicator registrations of this study were done using the tandem contours of CT and MR images, the maximum values of both AP shift and Lat Shift at the tip of tandem and the ring level were within 1 mm. The systematic errors due to rotational error along φ and ψ due to 6 cm long tandem were within 1° and hence the maximum impacts on DVH parameters for ψ and φ rotational errors were within 5% and 2% respectively. The maximum (average) absolute percent changes of dose per mm of D_0.1cc_ of bladder due to systematic errors along the lateral, cranio-caudal and antero-posterior directions were 6.62% (3.54%), 3.79% (0.86%), 10.11% (6.73%) respectively and for those changes due to the rotation (θ) about tandem was 5.59% (4.08%) per mm (figure 6a). In case of D_2 cc_, the maximum (average) absolute changes of dose per mm due to the errors along lateral, cranio-caudal and antero-posterior directions were 2.99% (1.22%), 1.41% (0.73%) and 5.75% (4.12%) respectively whereas the error of rotation (θ) about tandem results the maximum (average) value of 2.21% (1.04%) (figure 6b).

Figure 5b shows the linear variation of rectum DVH parameters (D_0.1cc_, D_1 cc_, D_2 cc_ and D_5 cc_) due to systematic translational (along cranio-caudal and antero-posterior directions) and rotational errors (along θ, φ and ψ) with respect to those of original reconstruction. Maximum ranges of variations from -18.59% to 26.6% and -16.11% to 21.89% were found for D_0.1cc_ of rectum due to cranio-caudal and Antero-posterior errors respectively whereas those due to lateral error were from -0.61% to 2.90%. When the rotational errors were introduced along θ, φ and ψ, both the errors along θ and φ produced the approximate linear changes of all DVH parameters (within the variation ranges from 0% to 4.04% and -1.45 to 6.8% respectively for D_2 cc_) whereas all the DVH parameters due to the error along ψ (rotation about x-axis) were found as non-linear changes (figure 5b). Maximum (average) absolute change of dose per mm for D_0.1cc_ and D_2 cc_ were found maximum as 3.69% (2.53%) and 2.82% (1.75%) respectively for the systematic error along cranio-caudal shift as compare to those of other systematic errors except φ and ψ rotational errors (figure 6a).

In case of sigmoid (figure 5c), all the DVH parameters varied linearly with systematic errors along lateral (-4.56% to 10.47%), cranial-caudal (-4.70% to 5.28%), AP shift (-16.10% to 27.65%) and θ rotation (-2.05% to 2.05%). D_1cc_ and D_5cc_ due to φ rotational error also varied linearly within the ranges from -12.5% to 42.95% and -8.74% to 19.01% respectively whereas D_0.1cc_ of sigmoid due to lateral error and all the DVH parameters due the rotational error along ψ varied in non linear pattern. D_0.1cc_ due to lateral errors varies upto 49.58% from those of original reconstruction geometry of applicator. Maximum (average) absolute percent change of dose per mm due to lateral error was 12.36% (4.09%) per mm for D_0.1cc_ whereas those of other DVH parameters were found within 3.28% (2.19%) per mm except those of error along φ and ψ (figure 6). All the DVH parameters of sigmoid were very sensitive to AP Shift, φ and ψ rotational errors.

Most of the DVH parameters of HRCTVs varied in non-linear pattern as shown in figure 5d. Maximum variation range from - 27.49% to 0.49% was found for AP shift error as compare to other ranges from -10.6% to 0.51% and -7.65% to 6.49% of Lat Shift and Cranio-caudal Shift errors respectively. In case of rotational error, φ and ψ rotational errors produced the variation in DVH parameters from -27.93% to 1.35% and -28.39% to 11.77% respectively. Percent absolute change of dose per mm for HRCTV D_98_ and D_90_ were within the maximum values of 8.39% per mm and 8.37% per mm and average values of 6.14% per mm and 5.51% per mm respectively. HRCTV V_90_ was not affected by the systematic errors, introduced in this study. Maximum changes of HRCTV V_90_ were found within 2.09%. The changes in DVH parameters calculated due to inter-observer variation of applicator reconstruction using rigid registration methods were also reported by Kari Tanderup et al.^15^ They reported the changes of DVH parameters of bladder and rectum by 5-6 % per mm displacement of applicator in ant-post direction. For the other directions and other DVH parameters, the changes were lesser than 4 % per mm. Our data were well congruent as that of the mean change of dose per mm of bladder and rectum which were within 4% per mm due to lateral and cranio-caudal shifts. We found a very large change of 10.92% per mm and 12.16% per mm for φ and ψ rotational errors in all DVH parameter of all OARs (D_0.1cc_, D_1 cc_, D_2 cc_ and D_5 cc_) and HRCTV (D_90_ and D_98_) as compare to other translational and rotational errors. It is reasonably correct that Tanderup et al^11^ report only the error along θ for rotational error. As the alignment was done using tandem contours of CT and MR images, the rotational errors along φ and ψ are not happened frequently. However, a small change of applicator registration error of 1 mm at the tip of tandem will results 1° error in φ and ψ rotation about an origin at the centre of the ring and hence a large change in all DVH parameters can be happened (figure 5 & 6). These can be taken care by checking and reporting the accuracy of applicator registration at the tip along AP Shift and Lat Shift within 1.0 mm action limit. Cranial Shift largely affects the DVH parameters of rectum only (Figure 6) as compare to bladder, sigmoid and HRCTV. Action limits of 1.0 mm to 2.0 mm will produce the increase in variation of dose of rectum from those of original reconstruction ranges from 6.7 % to 10.9%. The limitation of this study is that the impacts of registration error on the changes of DVH parameters were considered only for a single patient. These impacts will be better quantified if a large population of patient data were utilized.

1. **CONCLUSION**

To our knowledge, our study is one of the new procedures for reporting the registration errors of CT MR fusion using rigid registration method for applicator reconstruction and to analyse the impact of registration errors on DVH parameters in Image based Brachytherapy planning. Image based Brachytherapy planning requires an accurate definition of dwell position of radiation source with respect to the tips of the applicators on the MR images. Choosing the ridge between the lumens at the tip of the ring applicator as reference point for dwell position definition decreased the uncertainty of digitization of catheter. The applicator geometries of micro Selectron HDR brachytherapy can be successfully reconstructed in treatment planning system using the rigid registration of applicators of CT and MR images and the information of applicator geometries from auto-radiographs. The applicator registration of CT and MR images using the contours of tandem and neck of the ring decreased the rotational error about tandem axis. The reconstruction accuracy of applicators was achieved within the action limit of 1.5 mm in this CT MR Image fusion technique. We recommended a verification method of CT MR image fusion using applicator registration which consists of 6 steps of verification at 3 different sites in ring applicator set for a perfect fusion. Rotational errors along φ and ψ rotation angles which produced large changes in DVH parameters can be tackled using AP Shift and Lat Shift at the tip of tandem. The maximum shift was found along the tandem axis in this technique.

**REFERENCES**

1. Haie-Meder C, Potter R, Limbergen EV, et al. Recommendations from Gynaecological (GYN) GEC ESTRO Working Group* (I): Concepts and terms in 3D image based 3D treatment planning in cervix cancer Brachytherapy with emphasis on MRI assessment of GTV and CTV. Radiotherapy and Oncology. 2005; 74:235-45.
2. Nag S. Cardenes H, Chang S, et al. Proposed Guidelines for Image based intracavitary brachytherapy cervical carcinoma: Report from Image-Guided Brachytherapy working group. Int J Radiat Oncol Biol Phys. 2004; 60(4):1160-1172.
3. Wachter-Gerstner N, Wachter S, Reinstadler E, Fellner C, Knocke TH, Pötter R The impact of sectional imaging on dose escalation in endocavitary HDR brachytherapy of cervical cancer: results of a prospective comparative trial. Radiotherapy Oncology. 2003; 68:51-59.
4. Viswanathan AN, Dimopolous J, Kirisits C, Berger D, Potter R Computed Tomography versus Magnetic Resonance Imaging- based contouring in cervical cancer brachytherapy: Results of a prospective trials and preliminary guidelines for standardized contours. Int J Radiat Oncol Biol Phys. 2007; 68(2):491-498.
5. Perez-Calatayud J, Kuipers F, Bellester F, et al. Exclusive MRI based tandem and colpostats reconstruction in gynaecological Brachytherapy treatment planning. Radiotherapy Oncology 2009; 91:181-186.
6. Kirisits C, Pötter R, Lang S, Dimopoulos M, Wachter-Gerstner N, Georg D. Dose and volume parameters for MRI-based treatment planning in intracavitary brachtytherapy for cervical cancer. Radiotherapy and Oncology 2005; 62(3): 901-911.
7. Kirisits C, Lang S, Dimopoulous M, Berger D, Georg D, Pötter R. The Vienna applicator for combined Intracavitary and Interstitial brachytherapy of cervical cancer: Design, Application, Treatment planning and Dosemetric results. Radiotherapy and Oncology 2006; 65(2): 624-630.
8. Oinam AS, Goda JS, Dubey S, Bhardwaj A, Patel FD Modeling of Correlation Indices between bladder and Foley’s catheter balloon dose with CT-based planning using limited CT Slices in Intracavitary Brachytherapy for carcinoma of cervix. Journal of Cancer Research and Therapeutic 2008; 4(4):156-163.
9. Pelloski CE, Palmer M, Chronowski GM, Jhingran A, Horton J and Eifel PJ Comparison between CT-based volumetric calculations and ICRU reference-point estimates of radiation doses delivered to bladder and rectum during intracavitary radiotherapy for cervical cancer. Int J Radiation Oncology Biol Phys 2005; 62(1):131–137.
10. Hellebust TP, [Tanderup](http://www.iop.org/EJ/search_author?query2=Kari Tanderup&searchfield2=authors&journaltype=all&datetype=all&sort=date_cover&submit=1) K, [Bergstrand](http://www.iop.org/EJ/search_author?query2=Eva Stabell Bergstrand&searchfield2=authors&journaltype=all&datetype=all&sort=date_cover&submit=1) ES, [Knutsen](http://www.iop.org/EJ/search_author?query2=Bj%F8rn Helge Knutsen&searchfield2=authors&journaltype=all&datetype=all&sort=date_cover&submit=1) BH, Roislien J, [Olsen](http://www.iop.org/EJ/search_author?query2=Dag Rune Olsen&searchfield2=authors&journaltype=all&datetype=all&sort=date_cover&submit=1) DR Reconstruction of a ring applicator using CT imaging: impact of the reconstruction method and applicator orientation. Phys. Med. Biol. 2007; 52(16):4893-904.
11. Berger D, Dimopoulos J, Potter R, Kirisits C Direct reconstruction of the Vienna applicator on MR images. Radiotherapy and Oncology 2009; 93:347-351.
12. Leeuw A.D., Moerland M.A., Nomden C, Tersteeg R.H.A, Roesink J.M., Jürgen-liemk-Schulz I.M. Applicator reconstruction and applicator shifts in 3D MR-based PDR brachytherapy of cervical cancer. Radiotherapy and Oncology. 2009; 93: 341-346.
13. Krempien TP, Dauuber S, Hensley FW, Wannenmacher M, Harms W Image fusion of CT MRI data enables improved target volume definition in 3D brachytherapy planning. Brachytherapy 2003; 2:164-71.
14. Haack Søren, Neilsen Søren Kynde, Lindegaard JC, Gelineck J, Tanderup K Applicator reconstruction in MRI 3D image-base dose planning of brachytherapy of cervical cancer. Radiotherapy and Oncology 2009; 91:187-193.
15. Kari Tanderup, Hellebust TP, Lang S, Grandfelt J, Potter R, Lindergard JC, Kirisits C, Consequences of Random and Systematic uncertainties in 3D image based brachytherapy in cervical cancer. Radiotherapy and Oncology 2008; 89:156-163.
16. Nesvacil N, Pӧtter R, Sturdza A, Hegazy N, Federico M, Kirisits C, Adaptive image guided brachytherapy for cervical cancer: A combined MRI-/CT-planning technique with MRI only at first fraction. Radiotherapy and Oncology 2013; 107(1): 75-81.

**FIGURE LEGEND**

Figure 1: Auto radiographs of Nucletron ring applicators set for source positions identification. Figure 1a shows the distances of the first dwell position from the middle point of the ridge between the air channel trends (lumen of the applicator) at the tip of ring applicator as 0.71 cm. The deviation of radio-opaque dummy source from the centre of dwell position of radiation source shows as 0.11 cm. Inset figure shows the definition of first dwell position from the middle point of the ridge between the inner air channel and outer surface of applicator at the tip using the Full width at half of the maximum CT number of line profile drawn on a line passing through the centre (dwell position) of the source.

Figure 2: Verification of CT and MR image fusion of tandem and ring applicators on para-sagittal plane with the positions of 4 verification sites. The inset figure (1), (2) and (3) show the verification of tandem and ring on para-coronal planes using dummy source, Neck of ring on para-axial plane, and the projected contours of ring applicators, reconstructed on CT and MR images respectively.

Figure 3: (a) Catheters reconstructed with the introduction of translational errors (1 mm to 5 mm in the step of 1 mm) along tandem axis (x), horizontal axis (y) and vertical axis (z). Red color spheres represent the original dwell position of reconstructed applicators and Purple spheres represent the same dwell positions with 5 mm error introduced to original reconstructed catheters in horizontal axis (x). (b) Catheters reconstructed with the introduction of rotational errors (0° to 19.1° in the step of 3.28°) along θ. Red color spheres represent the original dwell position of reconstructed applicators and Purple spheres represent the same dwell positions with 19.1° error introduced to original reconstructed catheters in horizontal axis (x).

Figure 4: Variation of 6 verification parameters for CT and MR image fusions at three different sites - ring level, tip level of tandem and neck level of ring: (a) Using water dummy source of MR image (10 cases of CT and MR image fusions) and (b) Without water dummy source of MR image (24 cases of CT and MR image fusions).

Figure 5: Impact of registration errors on DVH parameters of different OARs and HRCTV: % change of DVH parameters of bladder (a), rectum (b), sigmoid (c) and HRCTV (d) due to translational and rotational errors respectively from those of original reconstruction.

Figure 6: Impact of registration errors in terms of % absolute changes of DVH parameters per mm for different OARs and HRCTV: (a) % absolute change of dose per mm to 0.1 cc volumes of all OARs and HRCTV D_90_ due to translational and rotational errors. (b) % absolute change of dose per mm to 2 cc volume of all OARs and HRCTV D_98_ due to translational and rotational errors.
